# Downregulation of the splicing regulator NSRP1 confers resistance to CDK4/6 inhibitors *via* activation of interferon signaling in breast cancer

**DOI:** 10.1016/j.jbc.2024.108070

**Published:** 2024-12-10

**Authors:** Shiyi Yu, Yue Si, Miao Xu, Ying Wang, Chengxu Liu, Caili Bi, Maoqiu Sun, Haibo Sun

**Affiliations:** 1 Institute of Translational Medicine, Medical College, Yangzhou University, Yangzhou, Jiangsu, China; 2 Jiangsu Key Laboratory of Experimental & Translational Non-Coding RNA Research, Yangzhou University, Yangzhou, Jiangsu, China; 3 Department of Thyroid and Breast Surgery, The Affiliated Hospital of Yangzhou University, Yangzhou University, Yangzhou, Jiangsu, China; 4 Department of Obstetrics and Gynecology, Haian Hospital of Traditional Chinese Medicine Affiliated to Nanjing University of Chinese Medicine, Nantong, Jiangsu, China

**Keywords:** cell cycle, spliceosome, breast cancer, interferon, immunosuppression, CDK4/6 inhibitor, NSRP1

## Abstract

The combination of CDK4/6 inhibitors (CDK4/6i) and endocrine therapy is the first-line therapy for ER+/Her2-breast cancer; however, the development of drug resistance limited the efficacy of the agents. Although activation of the IFN signaling pathway has been identified as a critical driver of intrinsic and acquired CDK4/6i resistance, it remains unknown how the IFN signaling pathway was activated in resistant cells. Here, we report that NSRP1, a regulator of alternative mRNA splicing is downregulated in CDK4/6i resistant breast cancer cells and contributes to CDK4/6i resistance by mediating alternative splicing of NSD2 mRNA and activation of the IFN signaling pathway. Knockdown of NSRP1 reduces CDK4/6i (palbociclib) sensitivity of MCF7 cells while overexpression of NSRP1 sensitizes MCF7-PalR cells towards palbociclib treatment. Mechanistically, RNA sequencing suggests that NSRP1 knockdown strongly activates the IFN signaling pathway in MCF7 cells and elevates the expression of most of the "IFN-related palbociclib-resistance Signature" (IRPS) genes. NSRP1 also regulates numerous alternative splicing (AS) events in breast cancer cells, several of which are key regulators of the IFN signaling pathway. Among them, the inclusion of NSD2 exon 2 is elevated upon NSRP1 knockdown. Our data further show that the inclusion of NSD2 exon 2 is increased in breast cancer and associated with the poor prognosis of patients. In addition, including NSD2 exon 2 elevates NSD2 protein expression to activate the IFN signaling pathway. This study unveils the critical role of NSRP1 in regulating the IFN signaling pathway and the CDK4/6i resistance, which could be a promising biomarker for predicting therapy response.

According to statistics in 2023, breast cancer is the most prevalent cancer type for women worldwide (1). Of the subtypes of breast cancer, ER+/Her2-breast cancer constitutes approximately more than half of cases (2). Although ER-targeted therapy has achieved great success in combating ER+/Her2-breast cancer, many patients developed endocrine resistance which eventually led to mortality (3). After decades of studies, novel therapeutic targets have been developed to conquer the resistance (4). Among these targets, the combination of CDK4/6i with ER-targeted agents has shown a remarkable improvement in prolonging the survival of ER + breast cancer patients compared with ER-targeted agents alone (5, 6). However, several patients still suffer from intrinsic and acquired resistance to the approved CDK4/6i (palbociclib, ribociclib, and abemaciclib) (7). Recent studies unveiled that the elevation of cell cycle regulators (such as CDK7, CDK6, and CCNE1), and activation of the interferon (IFN) pathway were critical for developing CDK4/6i resistance in breast cancer (8, 9, 10, 11). There is still an urgent need to unveil the molecular mechanisms for drug resistance.

Alternative splicing (AS) enables the production of multiple mRNAs from a single gene (12). Dysregulation of RNA splicing generates cancer-specific transcripts and is now recognized as a novel hallmark of cancer (13). Mechanistically, RNA-binding proteins (RBPs) modulate the organization of spliceosomes or transport of pre-mRNAs to spliceosomes, therefore, aberrant expression of RBPs is frequently behind the dysregulated AS events in drug-resistant breast cancer cells (14, 15). Previous studies have discovered several AS events were critical for the development of endocrine resistance in breast cancer (16, 17). Most recently, RNA binding protein PRMT5 has been identified as a CDK4/6i resistance driver in breast cancer *via* controlling intron retention of cell cycle-regulating genes (18). However, many RBPs and dysregulated AS events critical for the emergence of CDK4/6i resistance have not been discovered.

NSRP1 (Nuclear Speckle Splicing Regulatory Protein 1) was first discovered as a protein containing a coiled-coil domain located in the nuclear speck (19). It was further validated as a critical component of the ribonucleoprotein complex mediating alternative splicing *via* spliceosome (19). Recent studies have revealed the vital roles of NSRP1 in the immune system development and suppressing metastasis of triple-negative breast cancer (20, 21). These findings suggest the involvement of NSRP1 in regulating cancer immunology.

The interferon (IFN) pathway is originally known as antivirus immune response in mammalian cells (22). In the past, studies have found an association between inactivation of the IFN pathway and tumorigenesis (23). In recent years, however, accumulating studies have demonstrated a protumor function of the IFN pathway in cancer cells (24, 25, 26). In contrast to short-term IFNγ stimuli, chronic IFNγ stimulation activated the expression of immune inhibitory factors, leading to T cell exhaustion and tumor immune evasion (27). Based on the clinical samples and multiple established CDK4/6i resistant breast cancer cell lines, a previous study reported a close connection between activation of the IFN pathway and CDK4/6i resistance (11). Their study found many targets of the IFN pathway were excellent predictors of response to CDK4/6i, termed IFN-related palbociclib-resistance signature (IRPS) (11). It remains unknown how the IFN pathway is activated in the CDK4/6i resistant breast cancer.

In the current study, we identified NSRP1 as a downregulated RBP in CDK4/6i resistant breast cancer whose high expression was associated with good prognosis of patients with ER+ breast cancer. In breast cancer cells, silencing NSRP1 reduced sensitivity toward palbociclib while the overexpression of NSRP1 sensitized cells to palbociclib. Mechanistically, RNA-Seq revealed that the downregulation of NSRP1 strongly activated the IFN pathway in breast cancer cells *via* regulating exon skipping of several regulators of the IFN signaling. These data suggest NSRP1 is a promising biomarker for patients receiving CDK4/6i treatment.

## Results

### NSRP1 is downregulated in CDK4/6i resistant breast cancer

**Figure 1:**
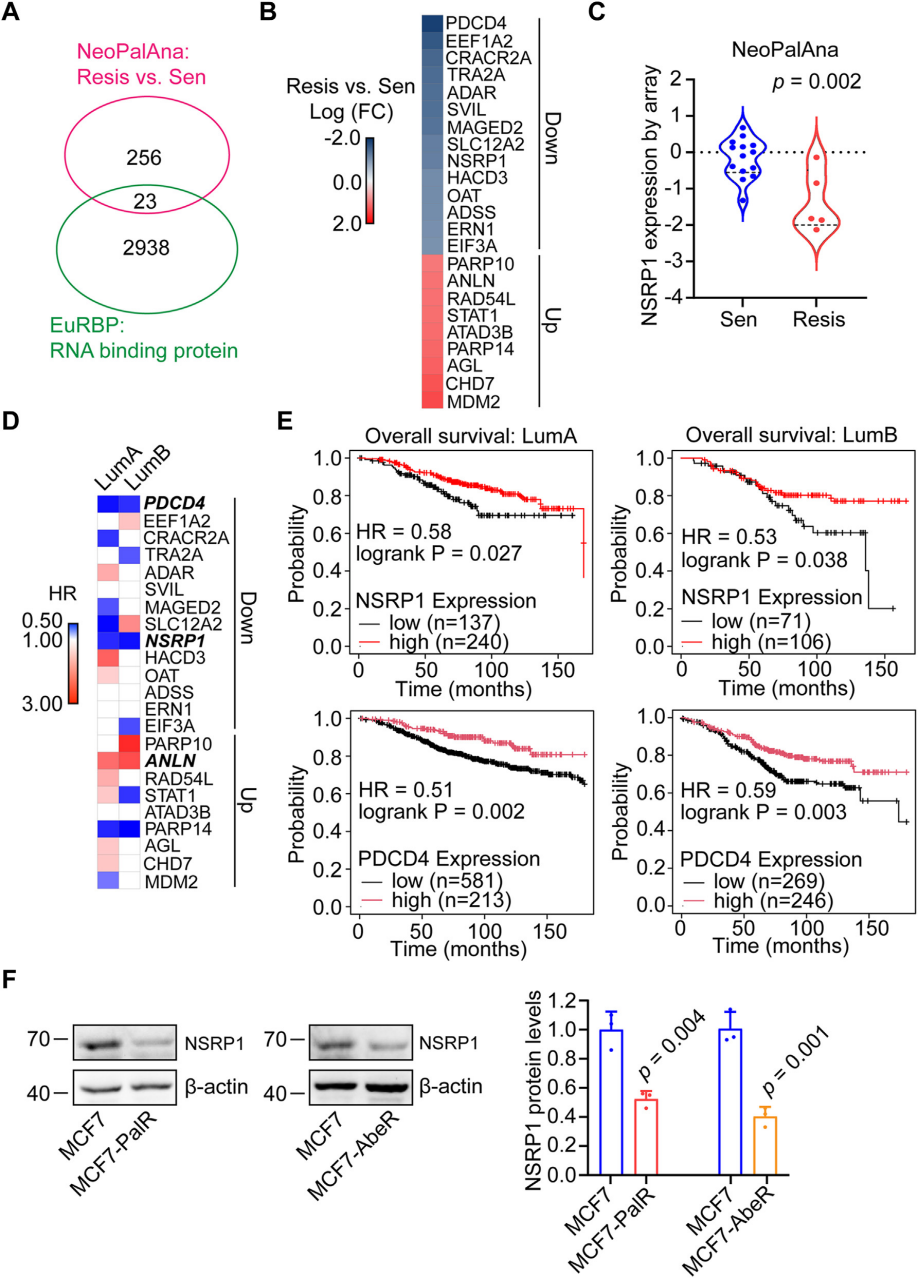
**NSRP1 was downregulated in CDK4/6i resistant breast cancer cells.***A*, overlap of significantly altered genes in CDK4/6i resistant ER+/Her2-breast tumors from the NeoPalAna project with RBPs from the EuRBP database. *B*, heatmap of the dysregulated RBPs in CDK4/6i resistant ER+/Her2-breast tumors. *C*, the expression of NSRP1 in CDK4/6i resistant (Resis) and sensitive (Sen) ER+/Her2-breast tumors from the NeoPalAna project. *D*, heatmap of the hazard ratio (HR) (overall survival, OS) between patients diagnosed with LumA or LumB breast cancer divided by high or low expression of the interested RBPs. Only the significant association was marked with color. *E*, Kaplan-Meier analysis of the association between NSRP1 or PDCD4 expression with OS of patients diagnosed with LumA or LumB breast cancer. *F*, Western blotting detection of NSRP1 protein expression in MCF7-AbeR, MCF7-PalR and MCF7 cells. Comparison by Student’s *t* test (*C*: n = 14 for Sen group, n = 5 for Resis group, unpaired; *F*: n = 3 replicates/group, unpaired) or Kaplan-Meier analysis (*D* and *E*). Error bars represent SD.

To explore the RNA-binding proteins (RBPs) involved in the development of CDK4/6i resistance, we analyzed microarray data of ER + breast cancer tissues from patients with palbociclib plus anastrozole (an endocrine therapy drug) treatment (NeoPalAna) (28). After that, the significantly dysregulated genes overlapped with the RBPs documented in the EuRBP database to identify key RBPs involved in resistance. 23 RBPs were significantly altered in tissues from patients sensitive to palbociclib plus anastrozole compared with those from patients resistant to palbociclib plus anastrozole (Fig. 1*A*). Most altered RBPs (14 out of 23) were downregulated in the tissues of patients resistant to palbociclib plus anastrozole, such as NSRP1 (Fig. 1, *B* and *C*). According to the clinical and expression data, 2 downregulated RBPs (PDCD4 and NSRP1) were significantly associated with prolonged overall survival of the patients with LumA and LumB breast cancer (Fig. 1, *D* and *E*). PDCD4 is a well-characterized tumor suppressor in breast cancer (29). Therefore, we focused on studying NSRP1. In two established CDK4/6i resistant breast cancer cell lines (MCF7-PalR and MCF7-AbeR) (Fig. S1, *A* and *B*), we observed downregulation of NSRP1 protein expression compared with the parental MCF7 cells (Fig. 1*F*). Interestingly, according to a previous RNA-Seq of breast cancer patients receiving endocrine therapy (30), no significant difference in NSRP1 expression was found between the sensitive and resistant groups (Fig. S1*C*). These data collectively implied that the loss of NSRP1 was a potential driver for resistance towards CDK4/6i plus endocrine therapy.

### NSRP1 negatively regulates CDK4/6i resistance in breast cancer cells

**Figure 2:**
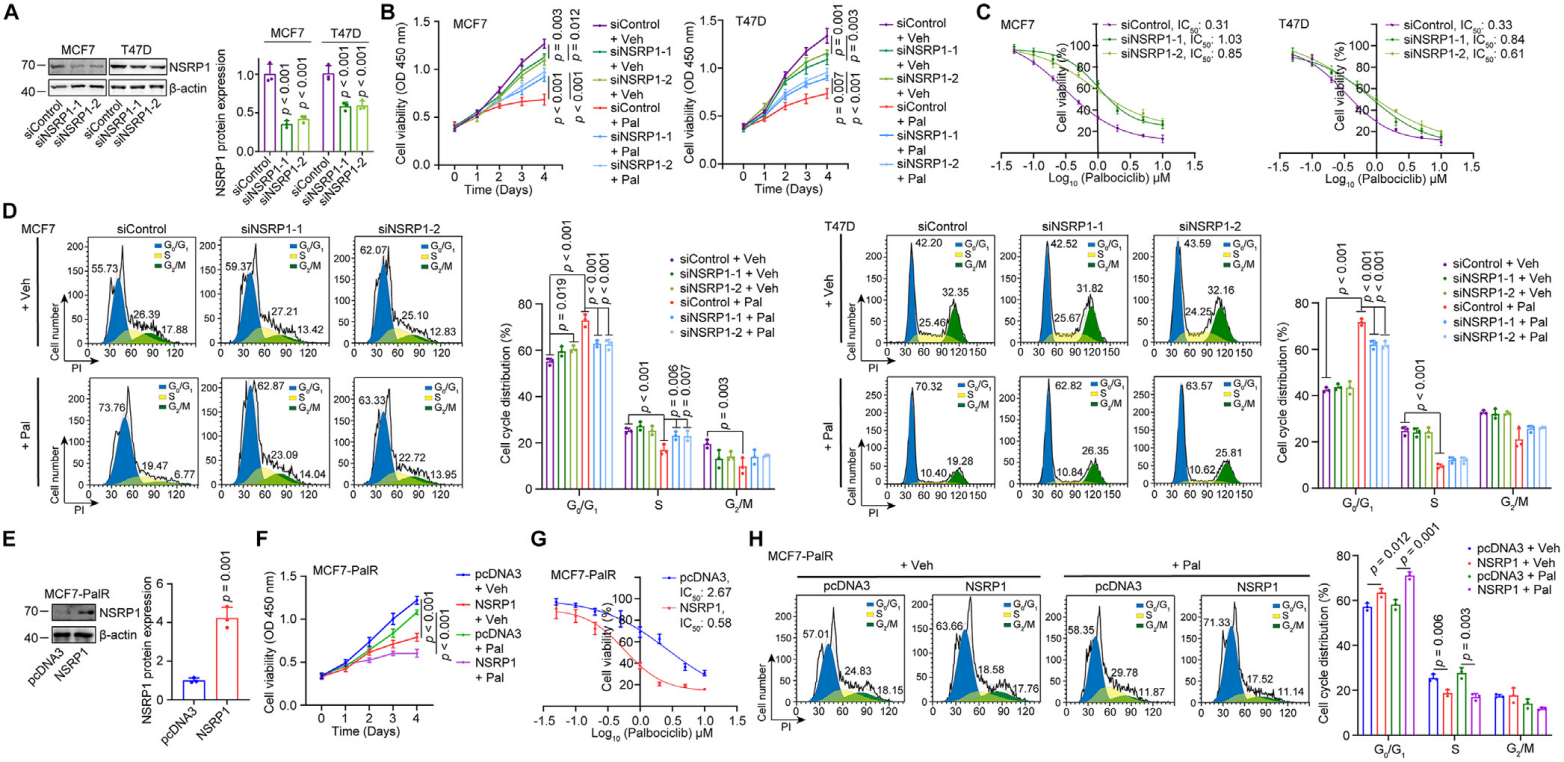
**NSRP1 expression was positively associated with palbociclib sensitivity in breast cancer cells**. *A*, Western blotting detection of NSRP1 protein expression in MCF7 or T47D cells with transfection of siNSRP1s or siControl. *B*, the CCK8 assay was performed to detect the cell proliferation ability of MCF7 or T47D cells with transfection of siNSRP1s or siControl and treated with or without palbociclib. *C*, after transfected with siNSRP1s or siControl, MCF7 or T47D cells were treated with increasing concentrations of palbociclib. The growth curves were depicted and the IC_50_ values were calculated. *D*, flow cytometry detection of cell cycle distribution of MCF7 or T47D cells with transfection of siNSRP1s or siControl and treated with or without palbociclib. *E*, Western blotting detection of NSRP1 protein expression in MCF7-PalR cells with transfection of pcDNA3 or pcDNA3-NSRP1. *F*, the CCK8 assay was performed to detect the cell proliferation ability of MCF7-PalR cells with transfection of pcDNA3 or pcDNA3-NSRP1 and treated with or without palbociclib. *G*, after transfected with pcDNA3 or pcDNA3-NSRP1, MCF7-PalR cells were treated with increasing concentrations of palbociclib. The growth curves were depicted and the IC_50_ values were calculated. *H*, flow cytometry detection of cell cycle distribution of MCF7-PalR cells with transfection of pcDNA3 or pcDNA3-NSRP1 and treated with or without palbociclib. Comparison by one-way ANOVA followed by the Tukey test (*A*–*D*, *F*, and *H*: n = 3 replicates/group) or Student’s *t* test (*E*: n = 3 replicates/group, unpaired). Error bars represent SD.

To study the function of NSRP1 on CDK4/6i resistance, we silenced NSRP1 in MCF7 and T47D cells (Fig. 2*A*). Downregulation of NSRP1 slightly inhibited cell proliferation, however, upon palbociclib treatment, the growth rate of NSRP1 downregulated cells was significantly higher than the control group for both MCF7 and T47D cells (Fig. 2*B*). Via treated with various concentrations of palbociclib, it was found that NSRP1 silencing MCF7 or T47D cells showed higher IC_50_ values towards palbociclib compared with the control group, suggesting NSRP1 silencing reduced the sensitivity of MCF7 and T47D cells to palbociclib (Fig. 2*C*). Flow cytometry analysis further revealed that palbociclib treatment induced cell cycle arrest at the G_0_/G_1_ phase in MCF7 and T47D cells, which was largely reversed in the NSRP1 silencing cells (Fig. 2*D*). However, NSRP1 knockdown showed no significant effect on cell proliferation of MCF7-PalR cells with or without palbociclib treatment which might be due to the low expression of NSRP1 (Fig. S2, *A* and *B*). Next, we overexpressed NSRP1 in the MCF7-PalR cells (Fig. 2*E*). Overexpression of NSRP1 suppressed cell growth and the growth rate of the NSRP1 overexpression cells was more significantly repressed by palbociclib compared with the control group (Fig. 2*F*). Moreover, NSRP1 overexpression cells showed lower palbociclib IC_50_ values than the control group, suggesting NSRP1 overexpression reversed palbociclib resistance of MCF7-PalR cells (Fig. 2*G*). Flow cytometry analysis also showed that NSRP1 overexpression cells showed a higher percentage of G_0_/G_1_ phase cells than the control cells upon palbociclib treatment (Fig. 2*H*). The data collectively showed that NSRP1 negatively regulated CDK4/6i resistance in breast cancer cells.

### Downregulation of NSRP1 activates the IFN pathway in breast cancer cells

**Figure 3:**
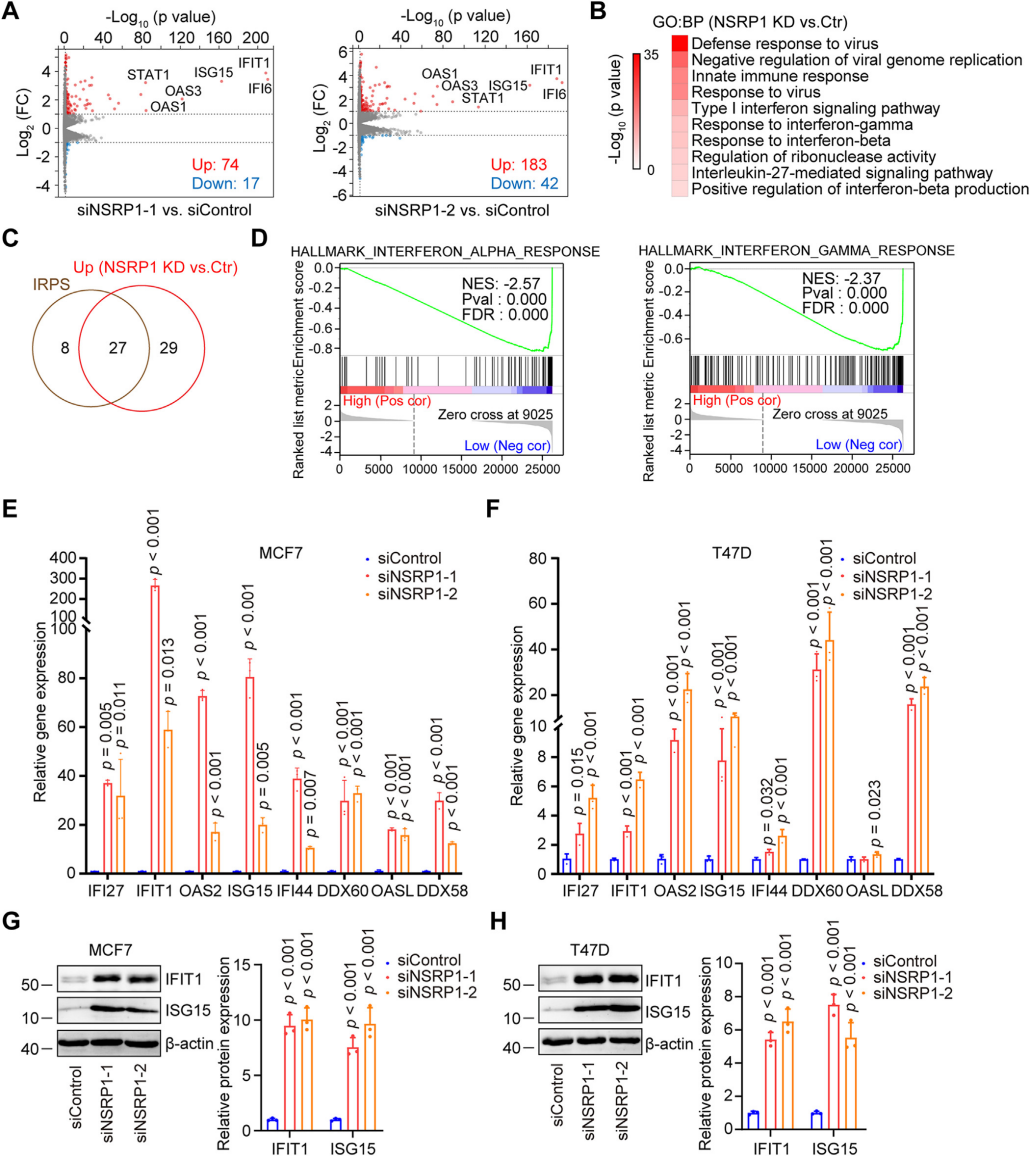
**NSRP1 silencing activated the IFN pathway**. *A*, Volcano plots of differentially expressed genes in MCF7 cells with transfection of siNSRP1s or siControl. *B*, GO:BP enrichment analysis of significantly altered genes (siNSRP1s vs. siControl). *C*, overlap of IFN-related palbociclib-resistance Signature (IRPS) genes with significantly upregulated genes (siNSRP1s *vs.* siControl). *D*, GSEA of HALLMARK gene set was applied to compare siNSRP1s and siControl. IFNα response and IFNγ response were the most significantly enriched and were presented. *E* and *F*, RT-qPCR detection of mRNA levels of IFN-stimulated genes in MCF7 (*E*) and T47D (*F*) cells with transfection of siNSRP1s or siControl. *G* and *H*, Western blotting detection of protein levels of IFN-stimulated genes in MCF7 (*G*) and T47D (*H*) cells with transfection of siNSRP1s or siControl. Comparison by one-way ANOVA followed by the Tukey test (*E*–*H*: n = 3 replicates/group). Error bars represent SD.

RNA-Seq was performed to analyze the transcriptome of NSRP1 silencing MCF7 cells and the control cells. It was observed that many IFN-stimulated genes were upregulated upon NSRP1 silencing in MCF7 cells (Fig. 3*A*). GO enrichment analysis showed that the upregulated genes of the NSRP1 silencing groups were mainly enriched in the IFN pathway (Fig. 3*B*). In addition, most of the upregulated genes were also included in the IFN-related palbociclib-resistance signature (IRPS) genes (Fig. 3*C*). Gene set enrichment analysis (GSEA) further identified the IFNα response and the IFNγ response pathways positively enriched in the NSRP1 silencing group (Fig. 3*D*). Interestingly, we found IFN-stimulated genes were strongly upregulated at mRNA (IFI27, IFIT1, OAS2, ISG15, IFI44, DDX60, OASL, DDX58) and protein (IFIT1 and ISG15) levels in MCF7-PalR and MCF7-AbeR cells compared with MCF7 cells (Fig. S3, *A* and *B*). Furthermore, short-term treatment of palbociclib or abemaciclib also elevated IFN-stimulated genes at both mRNA and protein levels, to a lesser extent, in MCF7 cells (Fig. S3, *C* and *D*). For validation, we performed RT-qPCR and the data showed that NSRP1 silencing strongly induced the elevation of many IFN-stimulated genes at mRNA levels in MCF7 and T47D cells (Fig. 3, *E* and *F*). The protein levels of IFIT1 and ISG15 were also strongly induced by NSRP1 silencing in MCF7 and T47D cells (Fig. 3, *G* and *H*). On the contrary, NSRP1 overexpression decreased mRNA and protein expression of these IFN-stimulated genes in MCF7-PalR cells (Fig. S3, *E* and *F*). To confirm the contribution of the IFN signaling to CDK4/6i resistance, we first treated MCF7 cells with IFNα2b to activate the IFN signaling in a dose-dependent manner (Fig. S3*G*). Consistently, it was observed that IFNα2b treatment desensitized MCF7 cells to palbociclib with higher IC_50_ values, especially in the high dose group (Fig. S3*H*), suggesting activation of the IFN signaling confers CDK4/6i resistance in breast cancer cells. Moreover, treatment of Ruxolitinib (an inhibitor of JAK/STAT and the IFN signaling) (31) decreased protein expression of ISGs (IFIT1, ISG15) in NSRP1 knockdown (NSRP1 si1) MCF7 cells (Fig. S3*I*), indicating inactivation of the IFN signaling. The cell viability assay further showed that Ruxolitinib treatment reduced the IC_50_ value of palbociclib in NSRP1 knockdown MCF7 cells (Fig. S3*J*). In addition, flow cytometry analysis showed that Ruxolitinib treatment induced a significant accumulation of NSRP1 knockdown MCF7 cells in the G0/G1 phase upon palbociclib exposure (Fig. S3*K*). These data showed that NSRP1 downregulation-induced activation of the IFN signaling was critical for CDK4/6i resistance development. Furthermore, by analyzing clinical samples, we observed a negative correlation between NSRP1 expression and the activity of the IFNγ pathway, in contrast to data from IRF3, a well-known transcription factor for the IFNγ pathway (Fig. S3*L*). These results suggested that NSRP1 downregulation activated the IFN pathway to confer CDK4/6i resistance in breast cancer cells.

### NSRP1 regulates AS events of key genes in the IFN pathway

**Figure 4:**
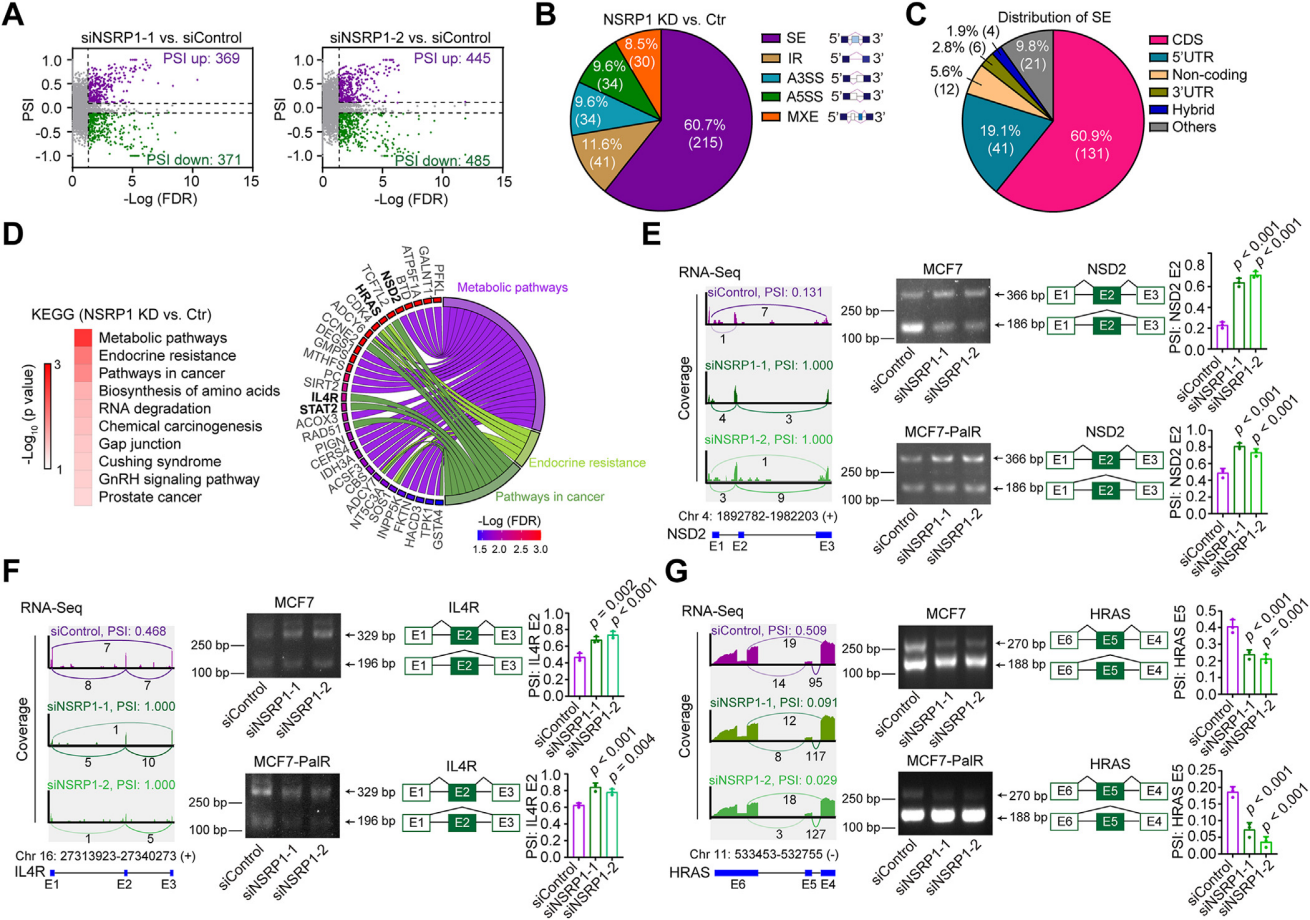
**NSRP1 regulated the AS of many oncogenes**. *A*, Volcano plots of significant altered AS events in MCF7 cells with transfection of siNSRP1s or siControl. *B*, Pie plot of the proportion of 5 AS types of significant altered AS events (siNSRP1s vs. siControl). *C*, distribution of AS events in locations of transcripts. *D*, KEGG enrichment of genes with altered AS events. The genes regulating the IFN pathway were labeled with bold style. *E–G*, Sashimi plots of altered AS events in NSD2, IL4R, and HRAS. RT-PCR was also performed to detect the inclusion of exons in NSD2, IL4R, and HRAS. Comparison by one-way ANOVA followed by the Tukey test (*E–G*: n = 3 replicates/group). Error bars represent SD.

NSRP1 has been reported to exert its function *via* controlling AS events. We next analyzed the AS events of the NSRP1 silencing and the control groups using the rMATS pipeline. Both NSRP1 siRNAs significantly altered around 800 AS events compared with the control group (Fig. 4*A*). For the AS events regulated by both NSRP1 siRNAs, most of them were skipping exon (SE, 60.7%), followed by intron retention (IR, 11.6%), alternative 3′ splice site (A3SS, 9.6%), alternative 5′ splice site (A5SS, 9.6%), and mutual exclusive exon (MXE, 8.5%) (Fig. 4*B*). The SE events were mostly distributed in the CDS region (60.9%) followed by the 5′UTR region (19.1%) (Fig. 4*C*). KEGG enrichment analysis showed that the genes with altered SE events upon NSRP1 silencing were involved in cancer-related processes, such as Metabolic Pathways, Endocrine Resistance, and Pathways in Cancer (Fig. 4*D*). Several reported regulators (NSD2, HRAS, IL4R, and STAT2) of the IFN pathways (32, 33, 34, 35) were also found in the list (Fig. 4*D*). Among them, the NSD2 exon 2 ranked top in differentially spliced exons (Table S1). For validation, we designed PCR primers to amplify the skipped exons. Consistent with the RNA-Seq data, the results showed that NSRP1 silencing promoted the inclusion of NSD2 exon 2 and IL4R exon2 while HRAS exon 5 was skipped upon NSRP1 knockdown (Fig. 4, *E*–*G*).

### The inclusion of NSD2 exon 2 is associated with the poor prognosis of patients with breast cancer

**Figure 5:**
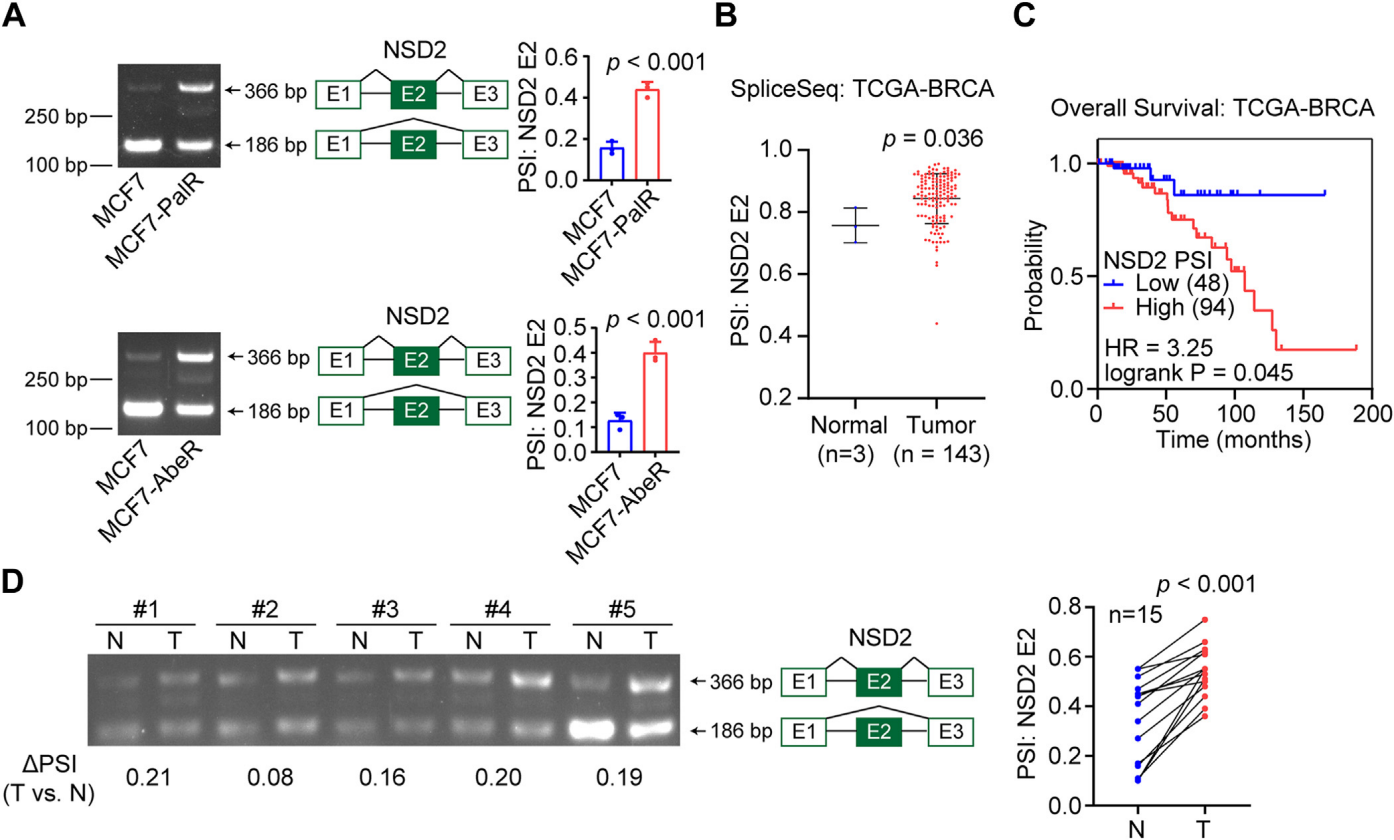
**The inclusion of NSD2 exon 2 was elevated in breast cancer**. *A*, RT-PCR detection of the inclusion of NSD2 exon 2 in MCF7 and MCF7-PalR cells. *B*, the PSI values of NSD2 exon 2 between breast tumors and normal tissues were compared. *C*, the association between the PSI values of NSD2 exon 2 and the overall survival of patients with breast cancer was analyzed with the Kaplan-Meier method. *D*, RT-PCR detection of the inclusion of NSD2 exon 2 in 15 pairs of breast tumors and matched normal tissues from patients diagnosed with ER+/Her2-breast cancer. Comparison by Student’s *t* test (*A*: n = 3 replicates/group, unpaired; *B*: n = 3 for Normal, n = 143 for Tumor, unpaired; D, n = 14 samples/group, paired) or Kaplan-Meier analysis (*C*). Error bars represent SD.

Compared with parental MCF7 cells, the inclusion of NSD2 exon 2 was increased in MCF7-PalR cells (Fig. 5*A*). According to the TCGA-BRCA dataset, the inclusion of NSD2 exon 2 was significantly elevated in the breast tumors compared with normal tissues (Fig. 5*B*). More importantly, the inclusion of NSD2 exon 2 was associated with poor overall survival of patients with breast cancer of various subtypes (Fig. 5*C*). We detected the status of NSD2 exon 2 in 15 ER + breast tumors and matched normal tissues. It was observed that the inclusion of NSD2 exon 2 was significantly elevated in our collected breast tumors compared with the counterparts (Fig. 5*D*). The data suggested that NSD2 exon 2 inclusion might generate oncogenic transcripts in breast cancer cells.

### The inclusion of NSD2 exon 2 elevates NSD2 protein expression and activates the IFN pathway

**Figure 6:**
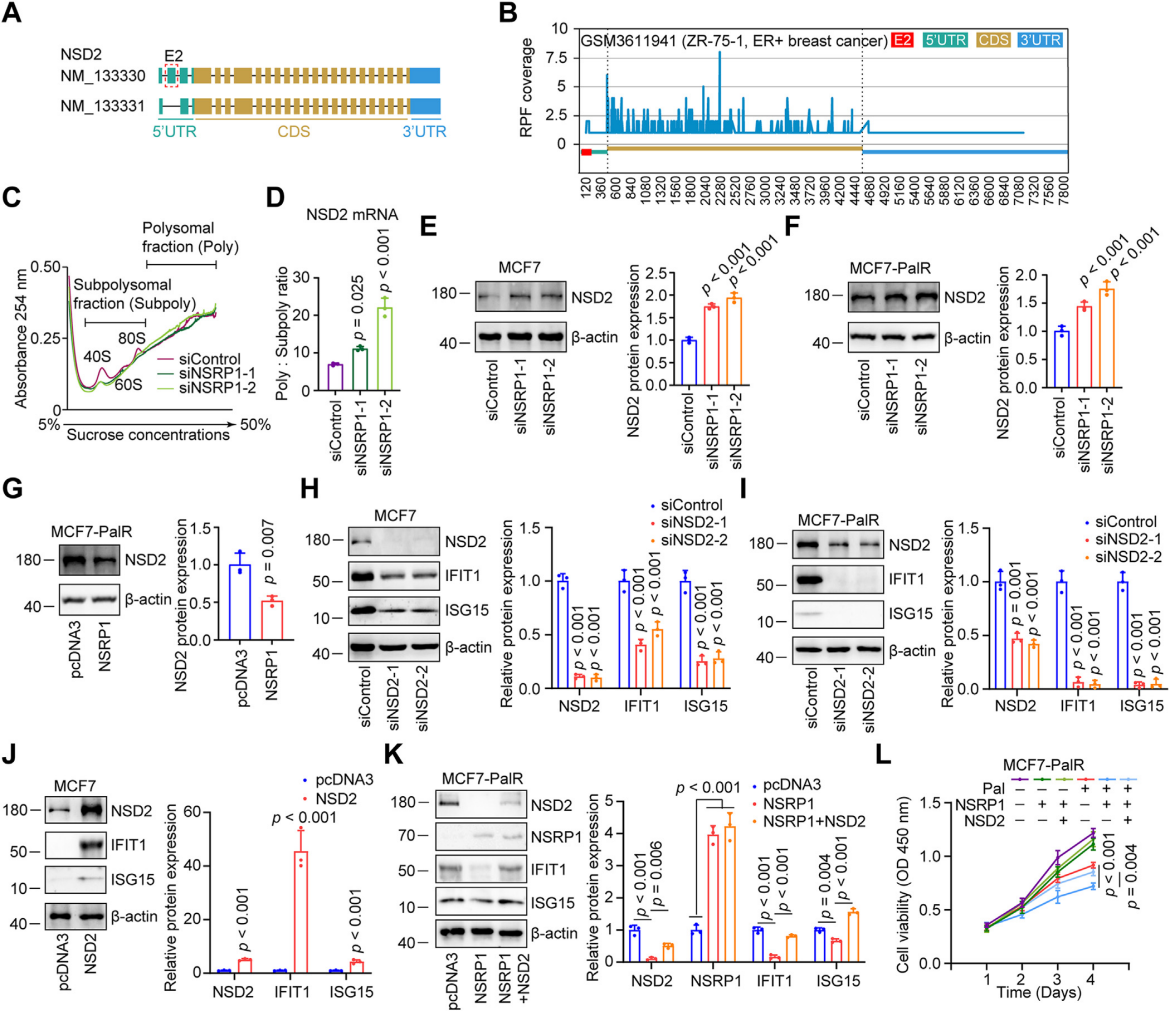
**NSRP1 negatively regulated NSD2 protein expression**. *A*, analysis of the structures of NSD2 transcripts with or without exon 2. *B*, ribo-Seq data showed that peaks were observed in the exon 2 region in the NSD2 transcript. *C*, the polysome profiling curves of siControl, siNSRP1-1, and siNSRP1-2 MCF7 cells were indicated. *D*, RT-qPCR was performed to detect the ratio of NSD2 mRNA in polysomal (Poly) and subpolysomal (Subpoly) fractions from polysome profiling of siControl, siNSRP1-1, and siNSRP1-2 MCF7 cells. *E* and *F*, Western blotting detection of protein levels of NSD2 in MCF7 (*E*) and MCF7-PalR (*F*) cells with transfection of siNSRP1s or siControl. *G*, Western blotting detection of protein levels of NSD2 in MCF7-PalR cells with transfection of pcDNA3 or pcDNA3-NSRP1. *H* and *I*, Western blotting detection of protein levels of NSD2 and IFN-stimulated genes in MCF7 or MCF7-PalR cells with transfection of siNSD2s or siControl. *J*, Western blotting detection of protein levels of NSD2 and IFN-stimulated genes in MCF7 cells with overexpression of NSD2. *K*, Western blotting detection of protein levels of NSRP1, NSD2, and IFN-stimulated genes in MCF7-PalR cells with overexpression of NSRP1 or NSD2 or NSRP1 + NSD2. Comparison by one-way ANOVA followed by the Tukey test (*D–F*, *H*, *I*, *K*, and *L*: n = 3 replicates/group) or Student’s *t* test (*G* and *J*: n = 3 replicates/group, unpaired). Error bars represent SD.

Via sequence analysis, we found that NSD2 exon 2 is located in the 5′UTR region of NSD2 transcripts (Fig. 6*A*). The inclusion of NSD2 exon 2 transformed transcript NM_133331 to transcript NM_133330 (Fig. 6*A*). According to the Ribo-Seq data, peaks were observed in the NSD2 exon 2 region in ZR-75-1 (ER+/Her2-), SUM159 (ER-/Her2-), and MCF10A (mammary epithelial) cells (Figs. 6*B* and S4, *A* and *B*), suggesting the sequence was involved in ribosome loading. Using polysome profiling, it was observed that the ratio of NSD2 mRNA in the subpolysomal fraction to the polysomal fraction (actively transcribed polysomes) in MCF7 cells was significantly elevated upon NSRP1 knockdown (Fig. 6, *C* and *D*), demonstrating that NSRP1 downregulation facilitated the translation of NSD2 mRNA. Consistently, western blotting showed that NSD2 protein levels were increased in NSRP1 silencing MCF7 cells compared with the control cells (Fig. 6*E*), however, the NSD2 protein stability was not altered upon NSRP1 silencing (Fig. S4*C*). In addition, NSD2 mRNA levels did not change significantly during NSRP1 silencing (Fig. S4*D*). These data suggested that the exon may be pivotal for translating the NSD2 mRNA. Similarly, NSRP1 knockdown elevated NSD2 protein expression in MCF7-PalR cells (Fig. 6*F*). On the contrary, NSRP1 overexpression decreased NSD2 protein expression without affecting NSD2 mRNA levels (Figs. 6*G* and S4*E*). NSD2 methylates H3K4 and H3K36 of the promoters, therefore facilitating the effect of IRF3 on stimulating the IFN pathway (32). It was found that NSD2 silencing decreased the protein expression of NSD2 and IFN-stimulated genes (IFIT1, ISG15) in MCF7 and MCF7-PalR cells (Fig. 6, *H* and *I*). The mRNA levels of IFN-stimulated genes were also reduced in MCF7 and MCF7-PalR cells upon NSD2 silencing (Fig. S4, *F* and *G*). In contrast, NSD2 overexpression elevated the protein and mRNA levels of IFN-stimulated genes in MCF7 cells (Figs. 6*J* and S4*H*). Most importantly, western blotting showed that NSD2 overexpression reversed the function of NSRP1 overexpression in suppressing the protein and mRNA expression of IFN-stimulated genes in MCF7-PalR cells (Figs. 6*K*, and S4*I*). Moreover, NSD2 overexpression also reversed the effects of NSRP1 overexpression in sensitizing MCF7-PalR cells to palbociclib treatment (Fig. 6*L*). These data implied that NSRP1 downregulation increased NSD2 expression to activate the IFN pathway *via* promoting the inclusion of NSD2 exon 2 in breast cancer cells.

### Low NSRP1 expression was associated with an immunosuppressive tumor microenvironment in ER+/Her2-breast cancer

**Figure 7:**
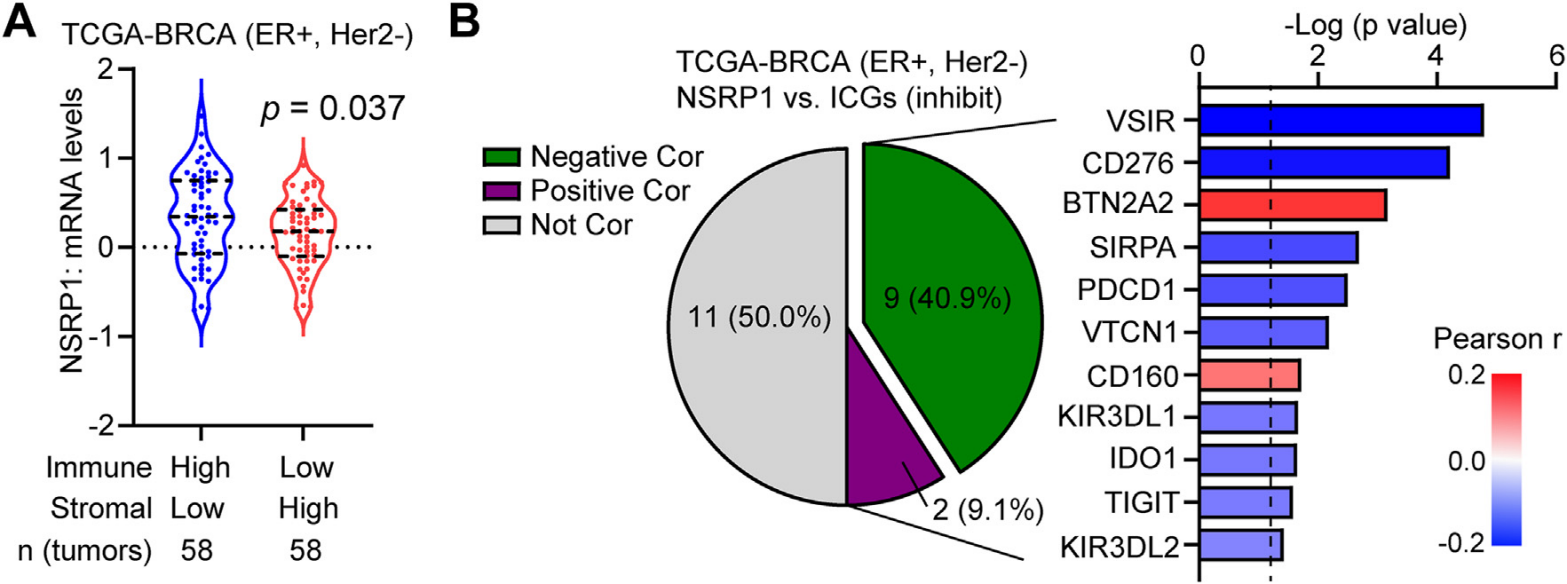
**Downregulation of NSRP1 was associated with the immunosuppressive microenvironment**. *A*, comparison of NSRP1 expression in Immune Score high/Stromal Score low ER+/Her2-tumors and Immune Score low/Stromal Score high ER+/Her2-tumors from the TCGA-BRCA dataset. *B*, the association between NSRP1 expression and the expression of inhibitory immune checkpoint genes in ER+/Her2-breast tumors from the TCGA-BRCA dataset. Comparison by Student’s *t* test (*A*: n = 58 tumors/group, unpaired) or Pearson correlation analysis (*B*). Error bars represent SD.

Because the infiltration of immune and stromal cells represents the tumor microenvironment in tumor tissues (36), we explored the association between the expression of NSRP1 and ESTIMATE scores (ESTIMATE Immune score and ESTIMATE Stromal score) in TCGA ER+/HER2- tumors. It was found that NSRP1 was lowly expressed in Immune score low/Stromal score high tumors compared with Immune score high/Stromal score low tumors (Fig. 7*A*), suggesting NSRP1 was associated with an immunosuppressive tumor microenvironment. Moreover, it was found that the expression of many inhibitory immune checkpoint genes was negatively correlated with NSRP1 levels, such as VSIR and CD276 (Fig. 7*B*). Collectively, these data manifested that ER+/Her2-breast tumors with low NSRP1 expression were potentially associated with an immunosuppressive tumor microenvironment, which might contribute to immune escape and poor prognosis.

## Discussion

**Figure 8:**
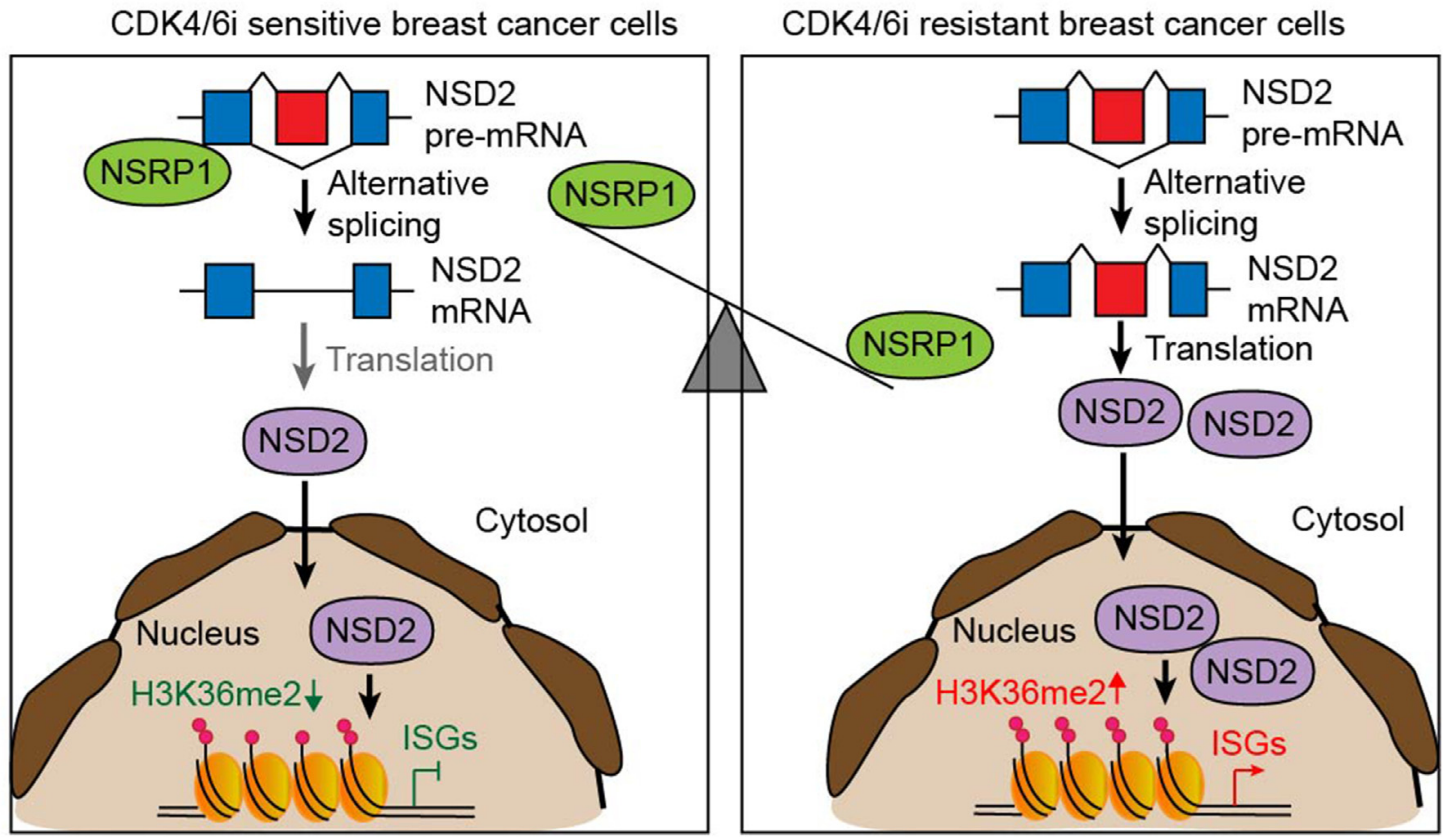
**Schematic representation of the role of NSRP1 in the regulation of CDK4/6i sensitivity in breast cancer cells**.

Although stimulation of the IFN signaling inhibited cell proliferation of breast cancer cells (37), chronic inherent activation of the IFN pathway is now recognized as a major trigger for chemotherapy resistance, radiotherapy resistance, tamoxifen resistance, and CDK4/6i resistance in breast cancer cells (11, 38, 39). Interestingly, our current study showed that NSRP1 downregulation not only inhibited cell proliferation of ER+ breast cancer cells but also conferred CDK4/6i resistance. These results indeed reveal the controversial role of INF signaling in breast cancer development. We further revealed that the downregulation of NSRP1 was responsible for the activation of the IFN pathway *via* regulating alternative mRNA splicing, suggesting NSRP1 was a promising biomarker for patients receiving CDK4/6i treatment (Fig. 8).

Most recently, it has been revealed that the expression of NSRP1 was decreased in subtypes of breast cancer and was associated with no recurrence and no metastasis status (21). In the current study, we observed a significant decrease in NSRP1 expression in palbociclib-resistant tumors and cell lines than their counterparts. In contrast to the previous report, which showed that NSRP1 expression was not associated with disease-free survival of Lum breast cancer (21), we observed that low expression of NSRP1 was significantly associated with poor overall survival of patients with LumA and LumB breast cancer. Our data also showed that NSRP1 downregulation conferred palbociclib resistance in ER + breast cancer cells and strongly alleviated cell cycle arrest in the G_0_/G_1_ phase upon palbociclib treatment. The findings manifested a tumor suppressor function of NSRP1 in ER+ breast cancer cells, providing novel insights in addition to the reported anti-metastasis function of NSRP1 in triple-negative breast cancer cells (21).

In 2011, NSRP1 was identified as a component of nuclear speckles, which are storage sites for pre-mRNA splicing machinery (19). NSRP1 reversed the splicing activity of oncogenic splicing factors SRSF2 and SRSF1 *via* physically interacting with the two splicing factors, regulating AS of CD44, Tra2β1, and Fas (19, 40). During thymocyte development, NSRP1 regulated AS of transcripts generated from cell cycle genes and SRSF1 (20). In triple-negative breast cancer, NSRP1 promoted the inclusion of NUMB exon 12 to antagonize epithelial-mesenchymal transition (EMT) *via* interaction with SRSF1 (21). Here, *via* RNA-Seq data analysis, we found NSRP1 regulated the splicing of pre-mRNAs transcribed from many oncogenes involved in metabolic pathways, endocrine resistance, and pathways in cancer in ER+ breast cancer cells. Among them, NSD2 methylated histones, facilitate the accessibility of IRF3 to IFN-I promoter and activate the IFN pathway (32). NSD2 could also methylate and activate STAT3 (41), which is a critical transcription factor for the IFN-stimulated genes (42). More importantly, targeting NSD2 enhanced the vulnerability of breast cancer cells to endocrine therapy (43). Our data showed that silencing of NSD2 also decreased the expression of the IFN-stimulated genes in ER + breast cancer cells, suggesting NSRP1-regulated AS of NSD2 might contribute to the activity of the IFN pathway. In detail, we found the inclusion or skipping of NSD2 exon 2, which was negatively regulated by NSRP1, generated 2 transcripts with different 5′UTR sequences. AS of 5′UTR determined the translatability of mRNAs in cancer cells (44). Consistent with the hypothesis, it was observed that NSRP1 silencing led to an increase of NSD2 transcripts in the subpolysomal fractions and elevation of NSD2 protein expression in ER+ breast cancer cells, suggesting that NSRP1 inhibited the translation of NSD2 *via* promoting the skipping of exon 2 from NSD2 pre-mRNA. The IFN pathway was associated with the immunosuppressive microenvironment in ER+ breast cancer and resistance to CDK4/6i (11). Interestingly, we also found that NSRP1 low-expression tumors exhibited high expression of inhibitory immune checkpoint genes. The results manifested that NSRP1 might be involved in the immunosuppressive microenvironment and this could partly explain the NSRP1-related CDK4/6i resistance in breast cancer cells. Therefore, the data collectively discovered an NSRP1/NSD2/IFN axis implicated in CDK4/6i resistance in ER+ breast cancer cells.

The study unveiled a critical role of NSRP1 downregulation in CDK4/6i resistance development, suggesting NSRP1 as a promising biomarker for patients with ER+ breast cancer. The data also identified AS events and the IFN pathway regulated by NSRP1 in breast cancer cells, providing novel insights into understanding the development of CDK4/6i resistance and optimizing treatment strategies for patients. Nonetheless, the current study is limited to providing direct evidence for the function of NSRP1 in immune suppression, which cannot be concluded that NSRP1 exerts its function *via* regulating immune cell infiltration directly in breast cancer cells. Therefore, a systematic study is needed to explore the function of NSRP1 in cancer immunology.

## Experimental procedures

### Human tissues

15 breast tumors and matched normal tissues were collected from patients diagnosed with ER+/Her2-breast cancer in the Affiliated Hospital of Yangzhou University from 2021 to 2023. None of the patients received chemotherapy or radiotherapy before the surgery. Written informed consents were collected from patients enrolled in the study. All experiments were approved and conducted under the supervision of the Ethic Committee of Yangzhou University (YXYLL-2021–08). Human studies in the current research abide by the principles of the Declaration of Helsinki. The samples were immediately transferred to liquid nitrogen for storage before extraction of RNA.

### Cell culture and reagents

ER+/Her2-breast cancer cell lines MCF7 and T47D were purchased from ATCC. These cells were cultured in RPMI1640 (Procell) supplemented with 10% FBS (HyClone) in a 37 °C incubator. The establishment of the MCF7-AbeR cell line was previously reported (3). The construction of the MCF7-PalR cell line was similar to MCF7-AbeR. Briefly, MCF7-TAMR cells were treated with increased concentrations of palbociclib (MCE) in combination with tamoxifen (MCE) for 6 months. After that, the CCK-8 assays were performed to determine palbociclib IC_50_ values of MCF7-TAMR and established MCF7-PalR cell lines. MCF7-AbeR and MCF7-PalR cells were maintained in RPMI1640 (no phenol-red) (Procell) with 10% FBS (activated charcoal absorbed) (VivaCell Biosciences) and abemaciclib (0.1 μmol/L) or palbociclib (0.5 μmol/L). The cytokine IFNα2b (MCE) was dissolved in water supplemented with 10% FBS. Ruxolitinib (Selleck), an inhibitor of JAK/STAT and the IFN signaling, was dissolved in DMSO and was used to inhibit the IFN signaling. To activate or inactivate the IFN signaling, cells were treated with IFNα2b (5000 or 10,000 Units/ml) or Ruxolitinib (10 μmol/L) for 1 day and then subjected to protein or RNA extraction.

### siRNA-mediated gene silencing

Control siRNA and siRNAs targeting NSRP1 or NSD2 were synthesized by GenePharma. Following the guideline of GP-transfect-Mate (GenePharma), siRNAs were diluted in OptiMEM (Invitrogen) and GP-transfect-Mate was then added to the mixture and maintained for 15 min. After that, the mixture was added to the cultured cells in the 6-well plates. 3 days later, the cells were harvested and the RNA and protein were extracted to detect the knockdown efficiency. The sequences of siRNAs were as follows: siControl: 5′-UUCUCCGAACGUGUCACGUTT-3′; siNSRP1-1: 5′-GCAGUGAGCAAGUUUGCAAAGTT-3′; siNSRP1-2: 5′-GAAACCCUCUAAUUCUGAAUCTT-3′; siNSD2-1: 5′-AGGGAUCGGAAGAGUCUUCAATT-3′; siNSD2-2: 5′-AACGGCCAGAACAAGCUCUUATT-3′.

### RNA sequencing

MCF7 cells were transfected with siControl or siNSRP1s. 3 days later, the cells were harvested and subjected to RNA extraction and library construction. The sequencing was conducted on a DNBSEQ platform (paired-end reads) by BGI. The sequencing raw data were filtered. The clean data was aligned to the Hg38 genome by the Hisat2 software, followed by processing with the Samtools and Featurecounts software sequentially. DeSeq2 was then used to compare the control group and the treatment group. Significantly differentially expressed genes were defined as log_2_fold change > 1 or < 0.5 and *p* < 0.05. The differentially expressed genes were enriched with the GO:BP using the DAVID software (https://david.ncifcrf.gov/tools.jsp). GSEA software was also used to study the association between altered transcriptome (siNSRP1 vs. siControl) with the HALLMARK gene sets (https://www.gsea-msigdb.org/gsea/msigdb/human/search.jsp).

To compare the AS event frequencies between the siNSRP1 group and the control group, we used the rMATS software (45) to process the Bam files generated from the clean data of RNA-Seq. PSI values (Percent Splice In) were acquired to manifest the occurrence of exons in transcripts. The AS events included 5 types, namely skipping exon (SE), intron retention (IR), alternative 3′ splice site (A3SS), alternative 5′ splice site (A5SS), and mutual exclusive exon (MXE). The significantly altered AS events were defined as PSI change > 0.1 or < −0.1 and FDR < 0.05. The genes with altered AS events were enriched with the KEGG by the DAVID software. The AS events were visualized with the IGV tool (46).

The data were deposited in the GEO database under the accession number GSE270673.

### Bioinformatic analysis

The NeoPalAna project is a phase II ER+/HER2-breast cancer clinical trial that collected tumor tissues before and after treatment of palbociclib in combination with anastrozole (a hormone therapy drug) and samples were subjected to microarray analysis. According to the Ki67 levels of post-treatment specimens, the patients were divided into palbociclib-sensitive (Ki67 ≤ 10%) and palbociclib-resistant (Ki67 > 10%) groups. The microarray data of the NeoPalAna project (17 and 5 cases sensitive and resistant to palbociclib respectively) was downloaded from the GEO database (GSE93204) (28). RNA-Seq data of primary pre-tamoxifen-treated and relapsed tamoxifen-resistant breast cancer tissues was acquired from the BioProject (PRJNA505938) (30). The list of RBPs was acquired from the EuRBP database (47). Kaplan-Meier software was used to investigate the association between overall survival and RBP expression in patients diagnosed with LumA or LumB breast cancer (Subtype-StGallen) (48).

TCGA SpliceSeq database (49) was used to retrieve PSI values for NSD2 exon 2 in 143 breast tumors and 3 normal tissues from the TCGA-BRCA project. The majority of these patients were ER+/Her2-breast cancer, and a third (43 out of 143) of these patients received endocrine therapy. cBioportal (https://www.cbioportal.org/) was used to obtain survival information of patients in the TCGA-BRCA project. Kaplan-Meier analysis was then performed to study the association between the PSI of NSD2 exon2 and the overall survival of patients with breast cancer of various subtypes. XENA (https://xenabrowser.net/) was used to acquire the activity of the IFNγ pathway in samples from the TCGA-BRCA dataset.

Ribo-Seq data were acquired from the Ribo-uORF database (50). We retrieved data from ZR-75-1, MCF10A, and SUM159 as representative cell lines.

The ESTIMATE (Estimation of STromal and Immune cells in MAlignant Tumor tissues using Expression data) Immune and Stromal scores of the TCGA-BRCA samples (ER+/Her2-breast tumors) were acquired from the ESTIMATE database (https://bioinformatics.mdanderson.org/estimate/index.html) (36). The list of inhibitory immune checkpoint genes was obtained from a previous study (51). The expression matrix of the inhibitory immune checkpoint genes and NSRP1 was downloaded from the TCGA-BRCA dataset using cBioportal. ER+/Her2-breast tumors were obtained from the TCGA-BRCA dataset, and the CYT and pDC scores of each sample were calculated according to a previous study (52). The infiltration of subpopulations of immune cells towards ER+/Her2-breast tumors was analyzed by the CIBERSORTx (53). The Pearson analysis was used to study the association between the expression of the inhibitory immune checkpoint genes, the infiltration of subpopulations of immune cells and the expression of NSRP1.

### NSRP1 overexpression

NSRP1 cDNA sequences were aligned to the pcDNA3 plasmid. pcDNA3-NSRP1 was transfected into cells with Lipo3000 (Invitrogen) to overexpress NSRP1 in indicated cells. 3 days later, the cells were harvested and the protein was extracted to confirm the overexpression efficacy.

### RNA extraction, RT-PCR, and RT-qPCR

TRIzol reagent (Invitrogen) was used to extract from cells and tissues. Tissues were cut into small pieces, added with TRIzol reagent, and rinsed in liquid nitrogen. The extracted RNA was quantified with a NanoDrop and reverse transcribed into first-strand cDNA with HiScript IV RT SuperMix for qPCR (Vazyme). RT-qPCR was then performed with ChamQ Universal SYBR qPCR Master Mix (Vazyme) following the manufacturer’s protocol. RT-PCR was performed to amplify the AS-related exons and the products were run on an agarose gel. The primer sequences are summarized in Table S2.

### Polysome profiling

After siRNA transfection, cells were cultured for an additional 48 h, then treated with cycloheximide (CHX) (Selleck) (0.1 mg/ml) for 2 min. After that, cells were harvested, lysed, and centrifuged to collect the supernatant. The supernatant was loaded onto a sucrose gradient, centrifuged with a Beckman SW41Ti rotor (38,000 rpm) at 4 °C, and fractioned according to the absorbance at 254 nm using an LC-UV100 equipment (WUFENG). The RNAs from the fractions were extracted using the TRIzol reagent for RT-qPCR.

### Cell proliferation and cell cycle analysis

The cell proliferation was detected using the CCK8 kit (Solarbio). Briefly, the CCK8 solution was mixed in the culture medium and the cells were incubated in the medium for 2 h. The absorbance (450 nm) was detected to reflect cell viability. To calculate IC_50_ values, cells were treated with increasing concentrations of palbociclib for 3 days. After that, the CCK8 assay was performed to measure the cell viability of each group, and the IC_50_ values were calculated by GraphPad Prism 8. To detect the distribution of cell cycle, cells were fixed in 70% ethanol overnight and stained with Propidium Iodide (PI) (Vazyme) for 30 min. The cells were then subjected to flow cytometry analysis. The data were analyzed with the Modfit software.

### Western blotting

Lysates were prepared from cells using the RIPA lysis buffer (Beyotime). The BCA kit was used to detect the concentrations of lysates. The lysates were run on an SDS-PAGE gel. The proteins were transferred to the PVDF membrane, and incubated with the primary and secondary antibodies sequentially. Finally, the membrane was developed with the ECL Western Blotting Substrate (Pierce; Thermo Fisher) and imaged with a ChemiDoc Imager (Shenhua Bio.). The intensity of bands was quantified using the ImageJ software and normalized to β-actin. The information on antibodies is listed in Table S3.

### CHX chase assay

The NSD2 protein stability was measured by the CHX chase assay. Briefly, NSRP1 silencing or control cells were plated in the 6-well plates and treated with CHX (20 μg/ml) for the indicated time. The cells were then harvested and subjected to western blotting.

### Statistical analysis

The data were analyzed with the GraphPad Prism 8 and showed as mean ± SD. All experiments were repeated thrice. Student’s *t* test was selected to compare 2 groups. Three groups were analyzed with one-way ANOVA followed by the Tukey test. *p* < 0.05 was considered statistically significant.

## Data availability

Other data supporting the study findings are available from the corresponding author upon reasonable request.

## Supporting information

This article contains supporting information.

## Conflict of interest

The authors declare no conflicts of interest with the contents of this article.
