# Supplementary material for: Downregulation of the splicing regulator NSRP1 confers resistance to CDK4/6 inhibitors via activation of interferon signaling in breast cancer
Source: J Biol Chem. 2024 Dec 10;301(1):108070. doi: 10.1016/j.jbc.2024.108070 (PMC11750474; doi:10.1016/j.jbc.2024.108070)
Supplement: Supporting information [file mmc1.docx]

**Supplementary Figure and Tables**

**
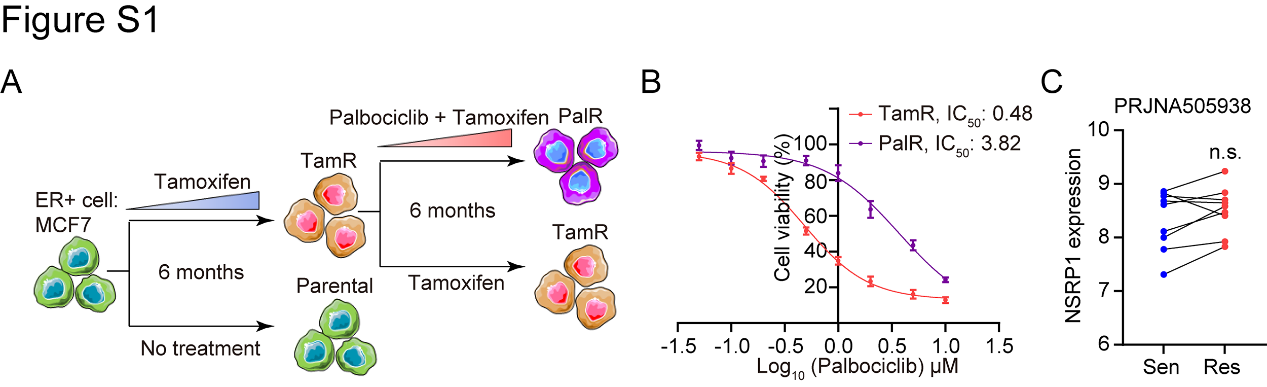
**

**Figure S1. Establishment of palbociclib resistant MCF7 cells (MCF7-PalR)**

A. A scheme of the procedure to establish MCF7-PalR cell. B. Drug response curves were plotted to study the palbociclib IC_50_ values of MCF7-TamR and MCF7-PalR cells. C. The expression of NSRP1 was compared between tamoxifen-sensitive and tamoxifen-resistant breast tumors from the dataset PRJNA505938 (n = 9 tumors/group, paired Student’s t-test). Error bars represent SD.


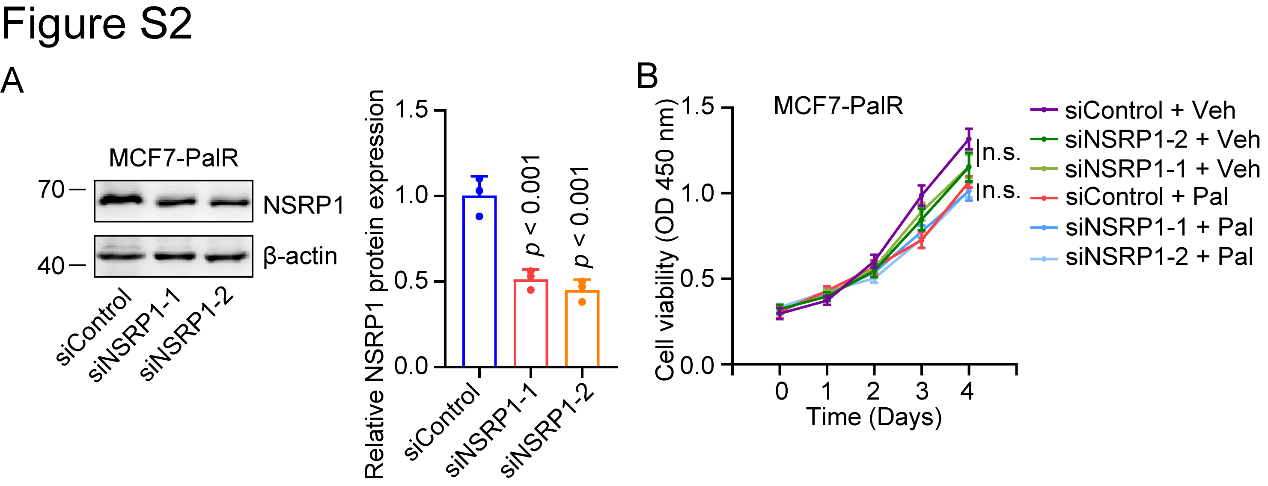


**Figure S2. Knockdown of NSRP1 showed no significant effect on palbociclib sensitivity of MCF7-PalR cells**

A. Western blotting detection of NSRP1 protein expression in MCF7-PalR cells transfected with siControl or siNSRP1-1 or siNSRP1-2. B. The growth curves of MCF7-PalR cells with or without NSRP1 knockdown treated with vehicle or palbociclib. Comparison by one-way ANOVA followed by the Tukey test (n = 3 replicates/group). Error bars represent SD.


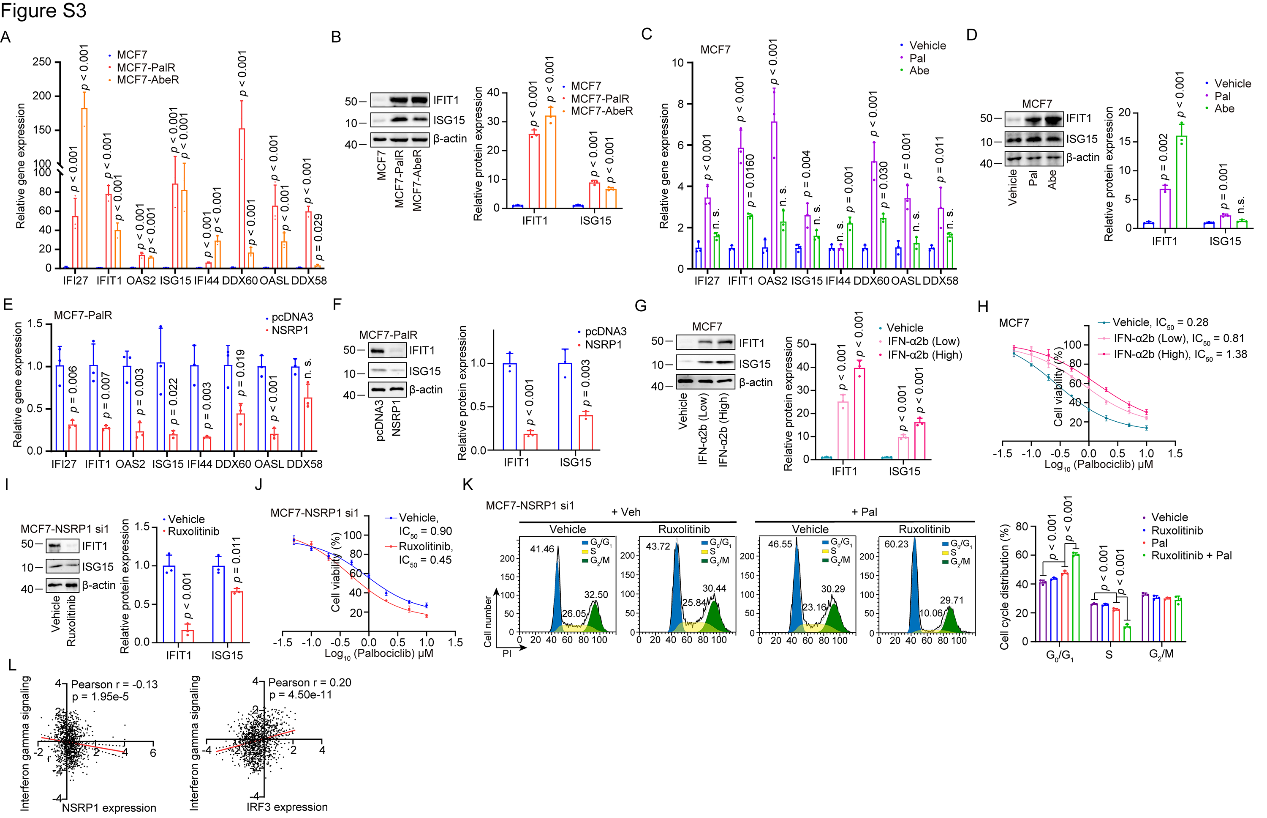


**Figure S3. NSRP1 negatively regulated the IFN pathway to increase palbociclib sensitivity in breast cancer cells**

A. RT-qPCR detection of mRNA levels of IFN-stimulated genes in MCF7, MCF7-PalR, and MCF7-AbeR cells. B. Western blotting detection of protein levels of IFN-stimulated genes in MCF7, MCF7-PalR, and MCF7-AbeR cells. C. RT-qPCR detection of mRNA levels of IFN-stimulated genes in MCF7 treated with vehicle, palbociclib, or abemaciclib. D. Western blotting detection of protein levels of IFN-stimulated genes in MCF7 treated with vehicle, palbociclib, or abemaciclib. E. RT-qPCR detection of mRNA levels of IFN-stimulated genes in MCF7 transfected with pcDNA3 or NSRP1 coding sequence. F. Western blotting detection of protein levels of IFN-stimulated genes in MCF7 transfected with pcDNA3 or NSRP1 coding sequence. G. Western blotting detection of protein levels of IFN-stimulated genes in MCF7 treated with vehicle or different doses of IFN-α2b. H. Drug response curves were plotted to study the palbociclib IC_50_ values of MCF7 cells treated with vehicle or different doses of IFN-α2b. I. Western blotting detection of protein levels of IFN-stimulated genes in MCF7-NSRP1 si1 cells treated with vehicle or Ruxolitinib. J. Drug response curves were plotted to study the palbociclib IC_50_ values of MCF7 cells treated with vehicle or Ruxolitinib. K. Flow cytometry detection of cell cycle distribution of MCF7-NSRP1 si1 cells with treatment of vehicle or Ruxolitinib and treated with or without palbociclib. L. Pearson correlation analysis of the correlation between NSRP1 and IRF3 expression with the activity of Interferon gamma signaling in samples from the TCGA-BRCA dataset. Comparison by one-way ANOVA followed by the Tukey test (A-D, G, K: n = 3 replicates/group) or unpaired Student’s t-test (F, I: n = 3 replicates/group). Error bars represent SD.


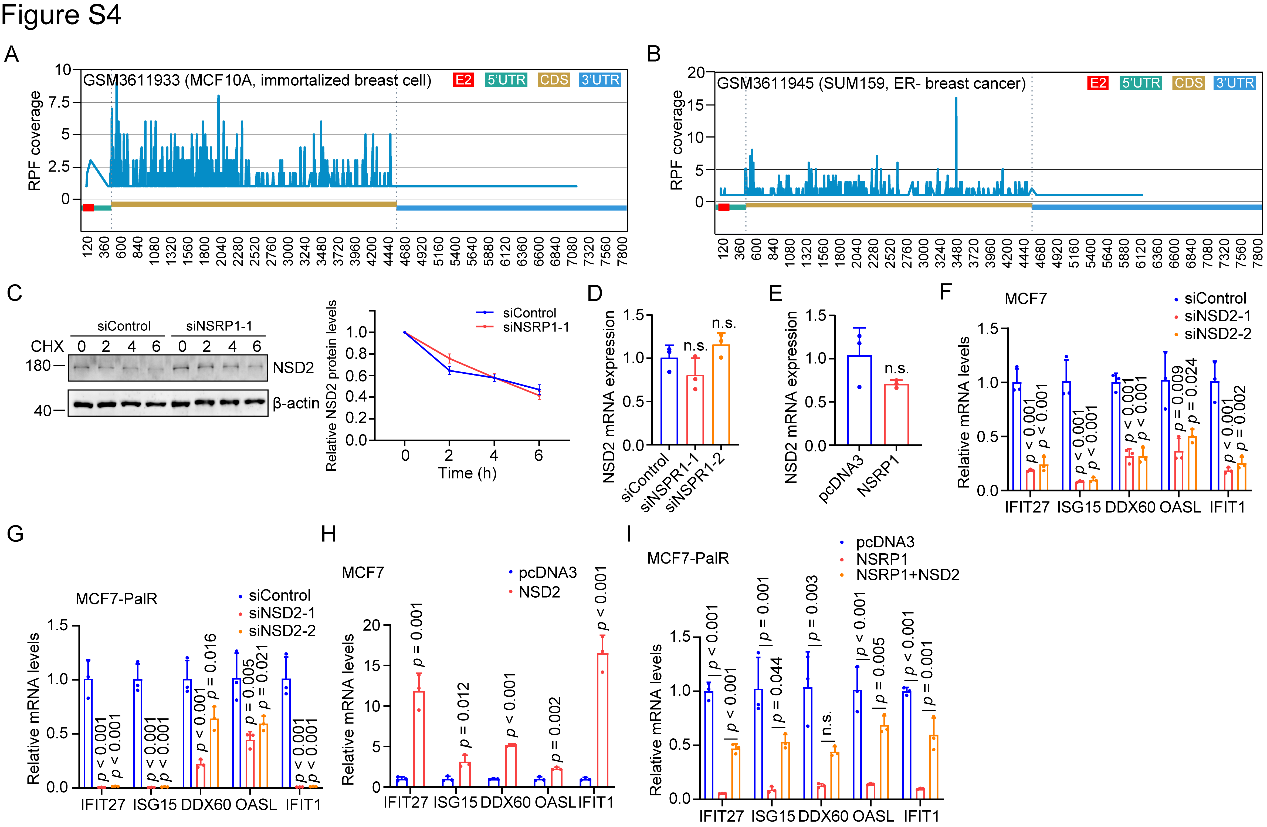


**Figure S4. Sequences encoded by NSD2 exon 2 are involved in ribosome loading in breast cancer cells**

A-B. Ribosomal profiling plots on the NSD2 transcript in MCF10A (A) and SUM159 (B) cells were retrieved from the Ribo-uORF database. C. MCF7 cells were transfected with siControl or siNSRP1-1, followed by treatment of CHX for indicated times (0, 2, 4, 6 h). The NSD2 protein expression in each group was then detected by western blotting. D-E. The NSD2 mRNA levels were detected in MCF7 cells with knockdown (D) or overexpression (E) of NSRP1. F-G. RT-qPCR detection of mRNA levels of IFN-stimulated genes in MCF7 (F) or MCF7-PalR (G) cells with or without NSD2 knockdown. H. RT-qPCR detection of mRNA levels of IFN-stimulated genes in MCF7 with or without overexpression of NSD2. I. RT-qPCR detection of mRNA levels of IFN-stimulated genes in MCF7-PalR cells transfected with pcDNA3 or NSRP1 coding sequence or NSRP1 coding sequence and NSD2 coding sequence. Comparison by one-way ANOVA followed by the Tukey test (D-F, G, I: n = 3 replicates/group) or unpaired Student’s t-test (E, H: n = 3 replicates/group). Error bars represent SD.

**Table S1. The list of altered exons involved in regulating the interferon pathway**

| **Gene** | **PSI (siNSRP1-1)** | **PSI (siNSRP1-2)** | **PSI (siControl)** | ***P* (siNSRP1-1 vs. siControl)** | ***P* (siNSRP1-2 vs. siControl)** |
| --- | --- | --- | --- | --- | --- |
| NSD2 | 1.0,0.833 | 1.0,1.0 | 0.266,0.131 | 6.35E-08 | 5.70E-09 |
| HRAS | 0.091,0.092 | 0.029,0.125, | 0.158,0.509 | 7.02E-04 | 3.94E-07 |
| STAT2 | 0.846,0.75 | 0.839,0.677 | 1.0,1.0 | 3.61E-04 | 8.85E-05 |
| IL4R | 1.0,1.0 | 1.0,0.787 | 0.346,0.468 | 4.85E-07 | 5.24E-05 |

**Table S2. The sequences of primers for RT-qPCR and RT-PCR**

| Name | Sequences |
| --- | --- |
| 18S-F | GTAACCCGTTGAACCCCATT |
| 18S-R | CCATCCAATCGGTAGTAGCG |
| IFI27-F | TGCTCTCACCTCATCAGCAGT |
| IFI27-R | CACAACTCCTCCAATCACAACT |
| IFIT1-F | TTGATGACGATGAAATGCCTGA |
| IFIT1-R | CAGGTCACCAGACTCCTCAC |
| OAS2-F | CTCAGAAGCTGGGTTGGTTTAT |
| OAS2-R | ACCATCTCGTCGATCAGTGTC |
| ISG15-F | CGCAGATCACCCAGAAGATCG |
| ISG15-R | TTCGTCGCATTTGTCCACCA |
| IFI44-F | ATGGCAGTGACAACTCGTTTG |
| IFI44-R | TCCTGGTAACTCTCTTCTGCATA |
| DDX60-F | CAGCTCCAATGAAATGGTGCC |
| DDX60-R | CTCAGGGGTTTATGAGAATGCC |
| OASL-F | CTGATGCAGGAACTGTATAGCAC |
| OASL-R | CACAGCGTCTAGCACCTCTT |
| DDX58-F | CTGGACCCTACCTACATCCTG |
| DDX58-R | GGCATCCAAAAAGCCACGG |
| NSD2-F | ACCGCGAGTGTTCTGTGTTC |
| NSD2-R | GTCGTGGCCGTTAAACTTCTG |
| NSD2-AS-F | CGTGCCTGTCCTAGAACCAC |
| NSD2-AS-R | CTGCTGCATCTCCTGCAAC |
| IL4R-AS-F | CTCCGCGCCCAGGAAAGC |
| IL4R-AS-R | CAGCTCACAGGGAACAGGAG |
| HRAS-AS-F | GCACGCACTGTGGAATCTC |
| HRAS-AS-R | CACAAGGGAGGCTGCTGAC |

**Table S3. The information on antibodies used in the study**

| Name | Company | Cat number | Dilution |
| --- | --- | --- | --- |
| anti-NSRP1 | Proteintech | 21360-1-AP | 1:1000 |
| anti-NSD2 | Proteintech | 22722-1-AP | 1:1000 |
| anti-β-actin | Proteintech | 66009-1-Ig | 1:1000 |
| anti-IFIT1 | Proteintech | 23247-1-AP | 1:1000 |
| anti-ISG15 | Beyotime | AF7305 | 1:1000 |
| HRP-conjugated goat anti-rabbit | Proteintech | SA00001-2 | 1:10000 |
| HRP-conjugated goat anti-mouse | Proteintech | SA00001-1 | 1:10000 |
